# AI Use, Perceptions, and Perceived Impact Among Nursing Students: Cross-Sectional Study

**DOI:** 10.2196/97832

**Published:** 2026-08-31

**Authors:** Ilda Taka, Elona Hasalla, Albana Sula, Blerina Bahiti, Rajmonda Oboni, Blerina Bani

**Affiliations:** 1 Department of Medical Technical Specialties, Faculty of Medical Technical Sciences, University of Elbasan, 3 Dëshmorët Street, Tirana, Albania, +355 673119890; 2 Department of Paraclinical Subjects, Faculty of Medical Technical Sciences, University of Elbasan, Elbasan, Albania; 3 Department of Nursing, Faculty of Medical Technical Sciences, University of Elbasan, Elbasan, Albania; 4 Department of Clinical Subjects, Faculty of Medical Technical Sciences, University of Elbasan, Elbasan, Albania

**Keywords:** artificial intelligence, nursing students, impact, perception, nursing education

## Abstract

**Background:**

AI is increasingly being integrated into education and health care, offering opportunities to improve learning, understanding of clinical cases, and students’ self-confidence. However, it remains necessary to assess nursing students’ perceptions of AI and its impact on their academic and professional development.

**Objective:**

The aim of the study was to assess AI use among nursing students, their perceptions of AI, and its impact on learning, academic performance, and professional preparation.

**Methods:**

A descriptive cross-sectional study with analytical components was conducted at the Faculty of Medical Technical Sciences in Elbasan, Albania. Data were collected through a structured questionnaire administered via Google Forms, which assessed AI use and its perceived impact on learning and professional preparation. Data were analyzed using SPSS (version 23.0). Descriptive statistics, chi-square tests for associations between variables (*P*<.05), and logistic regression to estimate crude odds ratios (CORs) and adjusted odds ratios (AORs) with 95% CIs were used.

**Results:**

A total of 279 nursing students participated in the study (mean age 22.4, SD 5.7 years), the majority of whom were female (273/279, 97.8%), lived in urban areas (156/279, 55.9%), and were enrolled in the bachelor’s program (198/279, 71%). Overall, 83.9% (234/279) reported using AI, mainly virtual assistants such as ChatGPT or similar tools (183/234, 78.2%). The most common reason was information searching (216/234, 92.3%), followed by studying and understanding lecture content (96/234, 41%). Bivariate analysis showed no significant associations between AI use and residence or study cycle, whereas grade point average (GPA) was significantly associated with AI use. In the multivariable analysis, GPA remained the only independent predictor of AI use. Students with a GPA of 6.0 to 6.9 (AOR 5.55, 95% CI 1.53-20.14; *P*=.009) and those with a GPA of 8.0 to 8.9 (AOR 5.73, 95% CI 1.26-26.00; *P*=.02) were significantly more likely to use AI than the reference group. Students perceived AI as having a moderate impact on learning, particularly understanding lectures (mean 2.58, SD 1.10) and exam preparation (mean 2.58, SD 1.02), whereas its impact on self-confidence (mean 1.97, SD 1.17) and empathy (mean 1.90, SD 1.12) was perceived as low. Although AI was considered useful for supporting learning (mean 2.83, SD 1.12), students expressed concerns regarding the reliability of AI-generated information (mean 3.18, SD 1.22), dependence on AI (mean 2.75, SD 1.28), and its impact on critical thinking (mean 2.80, SD 1.18).

**Conclusions:**

AI is widely used among nursing students, primarily supporting learning and academic performance. However, its impact on professional and interpersonal competencies remains limited. These findings suggest the need for integrating AI into nursing education curricula, with a focus on critical use and the development of students’ professional competencies.

## Introduction

In the last decade, AI has been one of the most important developments in the field of technology, and it has been integrated into various fields, including health care [1-3]. Its use is changing not only the way health care services are delivered but also the way future health care professionals are prepared, especially in the context of decision-making and patient management [4].

AI is also having a visible impact on nursing education. Today, students no longer rely solely on traditional learning methods but increasingly use digital methods to understand and assimilate their theoretical and practical knowledge [5]. AI-based technologies such as virtual simulations, virtual patients, and intelligent systems supporting decision-making have enabled an interactive and safe environment for the development of critical thinking and clinical reasoning [6,7]. These tools also contribute to the development of nursing care plans and an improved understanding of complex clinical processes [8].

Recently, a special role has also been played by various generative AI platforms such as ChatGPT and similar tools, which have become part of students’ daily lives [9]. These tools offer quick access to information, help explain difficult concepts, support the processing of academic materials, and generate structured responses similar to those produced by humans [10-12]. For nursing students, these technologies provide potential support for academic performance and professional development by linking theoretical, practical, and clinical knowledge [13].

Various studies have shown that the use of AI improves clinical reasoning abilities, critical thinking, and self-confidence in practice [1,14]. AI also provides personalized and interactive information, adapting to students’ needs and offering rapid feedback [15,16]. At the same time, the use of technology can help improve the quality of patient care through continuous support in monitoring and decision-making [17].

However, beyond its benefits, the literature shows that the use of AI is associated with a series of challenges and concerns in the field of nursing education. Uncontrolled use of AI may lead to reduced active student involvement in the learning process, increased dependence on technology, and reduced professional competence [18-20]. Other concerns relate to the accuracy of information, academic privacy, and the risk of plagiarism [21,22], issues that are particularly sensitive in the field of nursing.

Another aspect that should not be neglected is the need to develop new competencies among nursing students, including the critical use of technology and data analysis [23]. Importantly, AI cannot replace the human elements of the nursing profession such as empathy, communication, and patient care [24].

Although the use and impact of AI in nursing education continue to grow, evidence on how students use and perceive it in practice remains limited. Most studies focus on evaluating the theoretical benefits of AI, while less attention has been paid to students’ real-life experiences and the factors influencing their use of AI in the educational context [11]. Moreover, there are several barriers such as the lack of standardization in AI education and inequalities in access [25].

It is important to study nursing students’ perceptions and experiences regarding the use of AI tools, with a focus on identifying benefits, challenges, and implications to develop educational policies for the most effective and ethical use of AI [26,27].

Therefore, the aim of this study was to assess the use of AI among nursing students, their perceptions of AI, and the perceived impact of AI tools on the learning process, academic performance, and professional preparation.

## Methods

### Ethical Considerations

The study was not submitted for formal ethics review because it was conducted through an anonymous, voluntary, minimal-risk web-based survey and did not involve the collection of personally identifiable or sensitive personal information. Electronic informed consent was obtained from all participants before accessing the questionnaire. Participants were informed about the purpose of the study, voluntary participation, confidentiality, and anonymity. No personally identifiable information was collected, and participants could withdraw at any time by choosing not to complete the survey. All data were collected, stored, and analyzed anonymously in accordance with the general principles of research integrity outlined in the institutional framework [28], and no compensation was provided.

### Study Design and Setting

A descriptive cross-sectional study with analytical components was conducted among students enrolled in the General Nursing program at the Faculty of Medical Technical Sciences, University of Elbasan, Albania. The study was carried out between January 2026 and March 2026.

### Study Population and Sampling

The study included all students enrolled in the bachelor’s and master’s General Nursing programs. Students from other study programs and other universities were excluded. Participants were selected using convenience sampling, and participation was voluntary. The minimum sample size was calculated using the Cochran formula with a 95% CI, a 5% margin of error, and a total population of 1020 students. The calculation resulted in a minimum required sample size of 279 participants.

### Data Collection Procedure

Data were collected using a structured, self-administered questionnaire developed by the authors and distributed online through Google Forms (Alphabet Inc) in student communication groups. Participants were informed about the purpose of the study and were assured of the confidentiality and anonymity of their responses. Before completing the questionnaire, all participants provided electronic informed consent. Only fully completed questionnaires were included in the final analysis. Due to the method of questionnaire distribution, the exact number of students who received the survey link could not be determined.

### Study Instrument

The study instrument was developed by the authors based on the scientific literature regarding the use of AI in nursing education. The questionnaire was developed in Albanian and consisted of 3 sections.

The first section included demographic and academic information, including age, sex, place of residence (urban or rural), study cycle (bachelor’s or master’s), and academic grade point average (GPA). In the Albanian grading system, grades range from 4 to 10, with 10 representing the highest academic achievement. For analytical purposes, GPA was categorized into 5 groups: <6.0, 6.0 to 6.9, 7.0 to 7.9, 8.0 to 8.9, and 9.0 to 10.0. This section also included questions regarding AI use, frequency of use, types of AI tools used, and purposes of use. Questions related to AI tool types and purposes of use allowed multiple responses. AI tools were categorized as virtual assistants (ChatGPT [OpenAI], DeepSeek [DeepSeek AI], and Copilot [Microsoft Corp]), educational platforms (Learning Nurse; Steppingstones Partnership Inc), text-generation and text-enhancement tools (paraphrase tool), and other tools used for academic support and information retrieval. AI use was initially assessed using a screening question (yes or no). Only participants who reported using AI proceeded to the subsequent questionnaire sections, which assessed frequency of use, types of AI tools used, and the perceived impact of AI on learning and professional preparation. Participants who reported not using AI completed only the demographic and academic sections.

The second section assessed the perceived impact of AI on learning and professional preparation. This section included questions on AI’s impact on understanding lectures, exam preparation, academic performance, time saving during studying, understanding clinical cases, nursing care, self-confidence during clinical practice, critical thinking, communication skills, and empathy toward patients. All questions were assessed using a 5-point Likert scale ranging from 1 (“no influence”) to 5 (“very high influence”). For statistical analysis, these variables were grouped into 2 main dimensions: academic impact and professional impact.

The final section assessed students’ perceptions regarding the use of AI in nursing education. It included statements about the usefulness of AI, its impact on learning quality, the risk of dependence on technology, the reliability of AI-generated information, and its potential impact on critical thinking. Responses were recorded using a 5-point Likert scale ranging from 1 (“strongly disagree”) to 5 (“strongly agree”).

### Validity and Reliability

To evaluate the clarity, comprehensibility, and relevance of the questionnaire items in relation to the study objectives, the questionnaire was reviewed by 4 academic staff members with expertise in nursing education and research methodology. Their suggestions were considered, and minor modifications were made to the wording of several questions. Subsequently, a pilot test was conducted with 20 nursing students who had characteristics similar to those of the study population to assess comprehensibility, feasibility, and the time required to complete the questionnaire. On the basis of participants’ feedback, minor revisions were made to improve the instrument. Data collected during the pilot study were not included in the final analysis.

The internal consistency of the questionnaire was evaluated using the Cronbach α coefficient. The scale assessing the perceived impact of AI on learning outcomes (10 items) demonstrated excellent reliability (α=0.937). The scale assessing perceptions of AI use (6 items) demonstrated good reliability (α=0.810). These findings indicate satisfactory internal consistency for both scales, supporting their suitability for further statistical analyses.

### Data Analysis

Data were analyzed using SPSS (version 23.0; IBM Corp). Statistical analysis was conducted in several steps. First, descriptive statistics were used to summarize students’ sociodemographic and academic characteristics, as well as variables related to AI use. Categorical variables were presented as frequencies and percentages, whereas continuous variables were described using means and SDs. Variables measured using Likert scales were also analyzed descriptively and presented as frequencies and percentages for each response category, as well as means and SDs for each item.

The chi-square test was used to assess associations between AI use and categorical variables. Factors associated with AI use were further analyzed using logistic regression. Both crude odds ratios (CORs) and adjusted odds ratios (AORs), together with 95% CIs, were reported. A *P* value <.05 was considered statistically significant.

## Results

### General Characteristics

A total of 279 students enrolled in the General Nursing program participated in the study. Of these, 273 (97.8%) were female and 6 (2.2%) were male. The mean age of the participants was 22.4 (SD 5.7) years. More than half of the students (n=156, 55.9%) lived in urban areas, while 123 (44.1%) resided in rural areas. The majority of participants (n=198, 71%) were enrolled in the bachelor’s program. Regarding academic performance, 123 (44.1%) students had a GPA of 7.0 to 7.9, followed by 78 (28%) with a GPA of 6.0 to 6.9. Additionally, 45 (16.1%) students reported a GPA of 8.0 to 8.9 and 12 (4.3%) had a high GPA of 9.0 to 10.0. Students with a GPA of <6.0 accounted for 7.5% (n=21) of the participants. Overall, 234 (83.9%) students reported using AI tools (Table 1).

**Table 1.** Sample characteristics of participants.

Variables and categories	Participants
Age (y), mean (SD)	22.4 (5.7)
Residence, n (%)^a^	
Urban	156 (55.9)
Rural	123 (44.1)
Sex, n (%)^a^	
Male	6 (2.2)
Female	273 (97.8)
Study cycle, n (%)^a^	
Bachelor’s	198 (71)
Master’s	81 (29)
Academic average (grade point average), n (%)^a^	
<6.0	21 (7.5)
6.0-6.9	78 (28)
7.0-7.9	123 (44.1)
8.0-8.9	45 (16.1)
9.0-10.0	12 (4.3)
AI use, n (%)^a^	
Yes	234 (83.9)
No	45 (16.1)

a N=279.

### Use of AI

A high proportion of students (234/279, 83.9%) reported using AI. Among the 234 AI users, 105 (44.9%) reported daily use, while 69 (29.5%) used AI on a weekly basis. Less frequent use was reported by a smaller proportion of participants, with 20.5% (n=48) using AI rarely and 5.1% (n=12) using it once a month. Regarding the types of AI tools used, 183 (78.2%) participants reported using virtual assistants such as ChatGPT, DeepSeek, or Copilot. Educational platforms such as Learning Nurse were used by 63 (26.9%) students, while text-generation tools were reported by 36 (15.4%) participants. In addition, 21.8% (n=51) of participants reported using other AI tools. Regarding the purpose of AI use, the majority of students (n=216, 92.3%) used AI for information searching. Additionally, 41% (n=96) of participants used AI for studying and understanding lecture content, 16.7% (n=39) for academic assignments, and 12% (n=28) for understanding clinical cases (Table 2).

**Table 2.** Frequency, tools, and purpose of AI use.

Variables and categories	Participants, n (%)
Frequency of AI use^a^	
Every day	105 (44.9)
Every week	69 (29.5)
Every month	12 (5.1)
Rarely	48 (20.5)
Tools used^a^^,^^b^	
Virtual assistants (ChatGPT, DeepSeek, and Microsoft Copilot)	183 (78.2)
Educational platforms (Learning Nurse)	63 (26.9)
Text-generation tools (paraphrase tool)	36 (15.4)
Other	51 (21.8)
Purpose of AI use^a^^,^^b^	
Studying and learning lecture content	96 (41)
Academic assignments or essays	39 (16.7)
Understanding clinical cases	28 (12)
Information search	216 (92.3)

a N=234.

b Multiple-response questions; n summed in each section may be greater than N.

### Factors Associated With the Use of AI

Bivariate logistic regression analysis showed that place of residence and study cycle were not significantly associated with the use of AI. Although students living in urban areas had higher odds of using AI than those living in rural areas, this association was not statistically significant (COR 1.56, 95% CI 0.82-2.96; *P*=.23). Similarly, no statistically significant difference was observed between bachelor’s and master’s students (COR 0.84, 95% CI 0.42-1.65; *P*=.74).

In contrast, academic performance (GPA) was significantly associated with AI use. Compared with students with a GPA <6.0, those with GPAs of 6.0 to 6.9, 7.0 to 7.9, and 8.0 to 8.9 had higher odds of using AI, whereas no statistically significant association was observed for students with a GPA of 9.0 to 10.0.

In the multivariable analysis, place of residence and study cycle remained statistically nonsignificant. After adjusting for the variables included in the model, GPA remained the only independent factor associated with AI use. Students with a GPA of 6.0 to 6.9 (AOR 5.55, 95% CI 1.53-20.14; *P*=.009) and those with a GPA of 8.0 to 8.9 (AOR 5.73, 95% CI 1.26-26.00; *P*=.02) were approximately 5 times more likely to use AI than the reference group (GPA<6.0). The association observed for the GPA category of 7.0 to 7.9 in the bivariate analysis was no longer statistically significant after adjustment (AOR 1.69, 95% CI 0.59-4.85; *P*=.33), suggesting a possible influence of confounding factors. Similarly, no statistically significant association was found between AI use and the GPA category of 9.0 to 10.0 (AOR 0.51, 95% CI 0.11-2.33; *P*=.38; Table 3).

**Table 3.** Factors associated with the use of AI (N=279).

Variables and categories	AI use, n (%)	No AI use, n (%)	Crude odds ratio (95% CI)	*P* value	Adjusted odds ratio (95% CI)	*P* value
Residence						
Rural (n=123)	99 (80.5)	24 (19.5)	1.00	—^a^	1.00	—
Urban (n=156)	135 (86.5)	21 (13.5)	1.56 (0.82-2.96)	.23	1.44 (0.71-2.92)	.31
Study cycle						
Bachelor’s (n=195)	165 (84.6)	30 (15.4)	1.00	—	1.00	—
Master’s (n=84)	69 (82.1)	15 (17.9)	0.84 (0.42-1.65)	.74	1.31 (0.61-2.84)	.49
Academic average (grade point average)						
<6.0 (n=21)	10 (47.6)	11 (52.4)	1.00	—	1.00	—
6.0-6.9 (n=78)	66 (84.6)	12 (15.4)	6.05 (2.11-17.36)	<.001	5.55 (1.53-20.14)	.009
7.0-7.9 (n=123)	111 (90.2)	12 (9.8)	10.18 (3.59-28.88)	<.001	1.69 (0.59-4.85)	.33
8.0-8.9 (n=45)	39 (86.7)	6 (13.3)	7.15 (2.13-24.06)	.002	5.73 (1.26-26.00)	.02
9.0-10.0 (n=12)	8 (66.7)	4 (33.3)	2.20 (0.50-9.61)	.29	0.51 (0.11-2.33)	.38

a Not applicable.

### Students’ Perceptions of the Impact of AI

Participants generally reported a low to moderate impact of AI on learning outcomes, with mean scores ranging from 1.90 to 2.95 on a 5-point Likert scale. The highest perceived impact was reported for understanding clinical cases (mean 2.95, SD 1.20), followed by exam preparation (mean 2.58, SD 1.02) and understanding lectures (mean 2.58, SD 1.10), suggesting that AI plays a primarily supportive role in cognitive and academic processes. AI was also perceived as helpful in saving time during studying (mean 2.49, SD 1.22).

The perceived impact was lower in psychosocial and professional dimensions, including academic performance, critical thinking, communication skills, and nursing care. The lowest mean scores were reported for empathy toward patients (mean 1.90, SD 1.12) and self-confidence (mean 1.97, SD 1.17; Table 4).

**Table 4.** Impact of AI on learning outcomes (n=234).

Items	1: no influence, n (%)	2: low influence, n (%)	3: moderate influence, n (%)	4: high influence, n (%)	5: very high influence, n (%)	Scores, mean (SD)^a^
Understanding lectures	52 (22.2)	49 (20.9)	84 (35.9)	43 (18.4)	6 (2.6)	2.58 (1.10)
Exam preparation	45 (19.2)	54 (23.1)	93 (39.7)	39 (16.7)	3 (1.3)	2.58 (1.02)
Academic performance	81 (34.6)	54 (23.1)	81 (34.6)	15 (6.4)	3 (1.3)	2.17 (1.02)
Time saving while studying	60 (25.6)	66 (28.2)	60 (25.6)	30 (12.8)	18 (7.7)	2.49 (1.22)
Understanding clinical cases	36 (15.4)	39 (16.7)	87 (37.2)	45 (19.2)	27 (11.5)	2.95 (1.20)
Nursing care	87 (37.2)	39 (16.7)	78 (33.3)	21 (9)	9 (3.8)	2.26 (1.16)
Self-confidence	117 (50)	42 (18)	48 (20.5)	18 (7.7)	9 (3.8)	1.97 (1.17)
Critical thinking	84 (35.9)	57 (24.4)	69 (29.5)	15 (6.4)	9 (3.8)	2.18 (1.10)
Communication skills	99 (42.3)	39 (16.7)	60 (25.6)	24 (10.3)	12 (5.1)	2.19 (1.23)
Empathy toward patients	126 (53.8)	33 (14.1)	54 (23.1)	15 (6.4)	6 (2.6)	1.90 (1.12)

a Higher scores indicate greater perceived impact.

### Students’ Perceptions of AI Use

Students’ perceptions of the use of AI were generally characterized by a moderate level, with mean scores ranging from 2.45 to 3.18 on a 5-point Likert scale. The highest level of agreement was reported for concerns regarding the reliability of AI-generated information (mean 3.18, SD 1.22), reflecting a considerable degree of skepticism about its accuracy.

Overall, AI was perceived as a supportive tool for the learning process (mean 2.83, SD 1.12) and for improving the quality of studying (mean 2.77, SD 1.14), suggesting a generally neutral to slightly positive perception of its use in education. However, moderate concerns were also expressed regarding its impact on critical thinking (mean 2.80, SD 1.18) and the risk of developing a dependence on AI (mean 2.75, SD 1.28; Table 5).

**Table 5.** Students’ perceptions of AI use (n=234).

Statements	1: strongly disagree, n (%)	2: disagree, n (%)	3: neutral, n (%)	4: agree, n (%)	5: strongly agree, n (%)	Scores, mean (SD)^a^
AI is a useful tool.	39 (16.7)	35 (15)	102 (43.6)	40 (17.1)	18 (7.7)	2.83 (1.12)
AI improves the quality of learning.	39 (16.7)	45 (19.2)	84 (35.9)	51 (21.8)	15 (6.4)	2.77 (1.14)
AI helps in preparation for professional practice.	54 (23.1)	75 (32.1)	78 (33.3)	15 (6.4)	12 (5.1)	2.45 (1.08)
AI negatively affects critical thinking.	39 (16.7)	61 (26.1)	72 (30.8)	39 (16.7)	23 (9.8)	2.80 (1.18)
AI may create dependency.	66 (28.2)	27 (11.5)	75 (32.1)	42 (17.9)	24 (10.3)	2.75 (1.28)
Information from AI is not always reliable.	33 (14.1)	6 (2.6)	84 (35.9)	60 (25.6)	51 (21.8)	3.18 (1.22)

a Higher scores indicate stronger agreement with the statement.

## Discussion

### Principal Findings

This study identified a high level of AI use among nursing students, with 83.9% (234/279) reporting that they used AI tools. This finding is consistent with previous studies reporting the widespread use of digital technologies in higher education [29]. However, daily use was reported by only a proportion of students, suggesting that AI is used primarily as a functional tool for specific academic tasks and information retrieval rather than as a continuously integrated component of the learning process [30].

The results showed that the most commonly used AI tools were virtual assistants, particularly ChatGPT and similar applications (78.2%), reflecting a global trend toward the use of AI as a learning support tool. This finding is consistent with the existing literature, which suggests that AI facilitates access to information and supports the understanding of complex academic concepts [31-34]. However, the fact that AI was used mainly for information searching rather than for developing clinical skills or critical thinking suggests that students perceive AI primarily as an informational resource rather than an educational tool [9].

Overall, students perceived the impact of AI on both academic and professional outcomes as low to moderate. The greatest perceived impact was reported for understanding clinical cases and preparing for examinations, whereas the lowest perceived impact was reported for empathy toward patients, self-confidence, communication skills, and critical thinking. These findings suggest that AI is primarily perceived as a supportive tool for accessing and organizing information, while its role in developing interpersonal and professional competencies remains limited.

The greater perceived impact on understanding clinical cases may be attributed to the ability of AI tools to provide structured explanations, practical examples, and simplified clinical information. Similarly, the moderate perceived impact on understanding lectures and preparing for examinations suggests that students use AI as a complementary learning resource rather than as a transformative factor in academic performance [11,35,36].

Conversely, the limited perceived impact on critical thinking may reflect students’ uncertainty regarding the role of AI in developing analytical reasoning and reflective skills. Likewise, the low perceived impact on empathy and self-confidence suggests that students do not consider AI to be an important contributor to their professional development. These findings support the view that although AI can facilitate the learning process, it cannot replace the human dimensions of nursing practice, such as empathy, communication, and the nurse-patient relationship [37,38].

A considerable proportion of students also expressed concerns regarding the reliability of AI-generated information, the risk of dependence on AI, and its potential impact on critical thinking. These perceptions suggest that uncontrolled AI use may limit the development of clinical judgment and encourage reliance on ready-made answers without sufficient critical reflection [18,27].

At the same time, students reported positive perceptions regarding the benefits of AI in improving learning efficiency and facilitating access to information. This finding reflects a balance between the perceived benefits and risks of AI, with acceptance of the technology accompanied by awareness of its limitations [39-41]. These results suggest that nursing education is currently in a transitional phase of AI adoption, in which students recognize its potential while remaining cautious about its academic and professional implications.

Logistic regression analysis showed that academic performance (GPA) was the only independent factor associated with AI use. Students with GPAs ranging from 6.0 to 8.9 were more likely to use AI than those with lower GPAs. These findings suggest that students with better academic performance are more likely to integrate digital tools into their learning strategies, consistent with previous studies reporting a positive association between academic achievement and technology use in education [33,42].

However, the absence of a statistically significant association among students with the highest GPA (9.0-10.0) suggests that this relationship may not be linear. One possible explanation is that high-achieving students may be more selective and critical in their use of AI, preferring traditional learning methods. It should also be noted that the small number of participants in this subgroup may have reduced the statistical power of the analysis.

In contrast, place of residence was not significantly associated with AI use, suggesting relatively equal access to digital technologies among students from urban and rural areas. Similarly, no association was found between AI use and study cycle, indicating that AI use was comparable among both bachelor’s and master’s students. These findings suggest that AI use in this context is influenced more by individual factors, such as exposure to technology and personal attitudes toward innovation, than by the level of academic study [29,43].

Overall, the findings of this study indicate that AI is already widely integrated into the learning process of nursing students; however, its use remains primarily supportive and informational. Although students recognize its benefits for accessing and organizing information, important limitations remain regarding its use for developing clinical and professional competencies. These findings highlight the need for a more structured integration of AI into nursing curricula through educational approaches such as simulation-based learning, virtual clinical cases, and supervised AI-assisted learning activities, with the aim of strengthening students’ critical thinking, clinical reasoning, and professional competencies.

### Study Limitations

This study has several limitations. First, as the data were based on self-reported responses, the findings may be subject to reporting bias and subjectivity in students’ perceptions and evaluations of the impact of AI. Furthermore, the use of an online questionnaire may have favored participation by students who were more familiar with or interested in technology. In addition, the cross-sectional design and the conduct of the study at a single institution did not allow for the assessment of causal relationships, thereby limiting the generalizability of the findings. Therefore, future studies should use more comprehensive research approaches, including qualitative methods such as interviews, to gain a deeper understanding of nursing students’ perceptions and experiences regarding the use of AI. Moreover, multicenter studies with larger and more diverse samples are recommended to improve the representativeness and generalizability of the findings.

### Conclusions

The findings of this study showed that nursing students had a generally positive attitude toward the use of AI and perceived it as a supportive tool in the learning process. However, its impact was greater on theoretical aspects, whereas its impact on their professional and interpersonal skills was more limited. These results emphasize the need for a more advanced integration of AI into nursing education. Training, experience, the development of digital competencies, and a supportive academic environment are essential for these tools to be used effectively.

## Supplementary material

10.2196/97832 Checklist 1 CHERRIES checklist.

## Abbreviations

AOR: adjusted odds ratio
COR: crude odds ratio
GPA: grade point average
