# Supplementary material for: AI Use, Perceptions, and Perceived Impact Among Nursing Students: Cross-Sectional Study
Source: JMIR Nurs. 2026 Aug 31;9:e97832. doi: 10.2196/97832 (PMC13529259; doi:10.2196/97832)
Supplement: Supplementary file 1 [file nursing-v9-e97832-s001.docx]

**CHERRIES Checklist (Checklist for Reporting Results of Internet E-Surveys)**

**1. Design**

- Survey type: Descriptive cross-sectional web-based study with analytical components
- Mode of delivery: Online questionnaire using Google Forms
- Target population: Nursing students at the Faculty of Medical Technical Sciences, University of Elbasan, Albania
- Sampling method: Non-probability convenience sampling
- Recruitment method: The survey link was distributed through student communication groups

**2. Ethics and Consent**

- The study did not obtain formal ethical approval from an institutional review board prior to data collection.
- The study involved an anonymous, voluntary, web-based survey with no collection of personally identifiable information.
- Electronic informed consent was obtained from all participants before accessing the questionnaire.
- Participants were informed about the study purpose, voluntary participation, confidentiality, and anonymity.
- Participants could withdraw at any time by not completing the survey.
- All data were collected, stored, and analyzed anonymously.

**3. Development and Pre-testing**

- The questionnaire was developed by the research team based on relevant literature and study objectives.
- Content validity and clarity were reviewed by four academic experts in nursing and research methodology.
- A pilot test was conducted with 20 nursing students to assess clarity, feasibility, and completion time.
- Minor revisions were made based on feedback from the pilot study.
- Pilot data were excluded from the final analysis.

**4. Recruitment Process and Sample**

- Data collection period: 10 January – 30 March 2026
- The survey was distributed electronically via student communication groups (e.g., online class groups).
- A total of 279 responses were received.
- The number of individuals exposed to the survey invitation is unknown due to the distribution method.
- No incentives were provided for participation.

**5. Survey Administration**

- The survey was administered using Google Forms.
- A screening question on AI use (Yes/No) was included at the beginning of the questionnaire.
- Participants who reported AI use completed all relevant sections of the questionnaire, while non-users completed only demographic and academic sections.
- All questions were set as required, resulting in no missing data.
- No incomplete questionnaires were recorded.

**6. Response Rates**

- A total of 279 completed responses were included in the final analysis.
- Completion rate among submitted responses was 100%.
- The number of individuals who accessed the survey link is unknown due to the distribution method.

**7. Preventing Multiple Entries**

- The survey platform (Google Forms) was configured to allow one response per participant.
- No additional technical measures such as IP tracking or cookies were used.

**8. Data Handling and Analysis**

- All responses (n = 279) were included in the final dataset.
- No responses were excluded from the analysis.
- Data were exported from Google Forms and analyzed using IBM SPSS version 23.0 as described in the manuscript.
- Descriptive statistics, chi-square test, and logistic regression analysis (COR and AOR with 95% CI) were performed.

**9. Other Information**

- No financial incentives or rewards were provided.
- Participation was voluntary and anonymous.
- Data confidentiality and privacy were strictly maintained throughout the study process.
